# Recent Advances of DprE1 Inhibitors against *Mycobacterium tuberculosis*: Computational Analysis of Physicochemical and ADMET Properties

**DOI:** 10.1021/acsomega.2c05307

**Published:** 2022-11-03

**Authors:** Patrícia S. M. Amado, Christopher Woodley, Maria L. S. Cristiano, Paul M. O’Neill

**Affiliations:** † Center of Marine Sciences - CCMAR, University of Algarve, P-8005-039 Faro, Portugal; ‡ Department of Chemistry and Pharmacy, FCT, University of Algarve, P-8005-039 Faro, Portugal; § Department of Chemistry, University of Liverpool, Liverpool L69 7ZD, United Kingdom

## Abstract

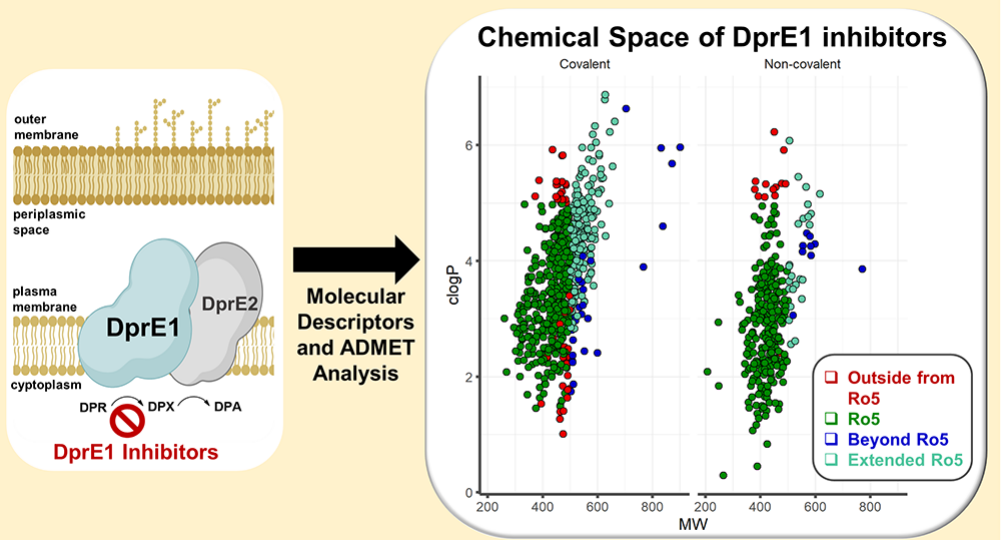

Decaprenylphosphoryl-β-d-ribose 2′-epimerase (DprE1) is a critical flavoenzyme in *Mycobacterium tuberculosis*, catalyzing a vital step in the production of lipoarabinomannan and arabinogalactan, both of which are essential for cell wall biosynthesis. Due to its periplasmic localization, DprE1 is a susceptible target, and several compounds with diverse scaffolds have been discovered that inhibit this enzyme, covalently or noncovalently. We evaluated a total of ∼1519 DprE1 inhibitors disclosed in the literature from 2009 to April 2022 by performing an in-depth analysis of physicochemical descriptors and absorption, distribution, metabolism, excretion, and toxicity (ADMET), to gain new insights into these properties in DprE1 inhibitors. Several molecular properties that should facilitate the design and optimization of future DprE1 inhibitors are described, allowing for the development of improved analogues targeting *M. tuberculosis*.

## Introduction

**Figure 1:**
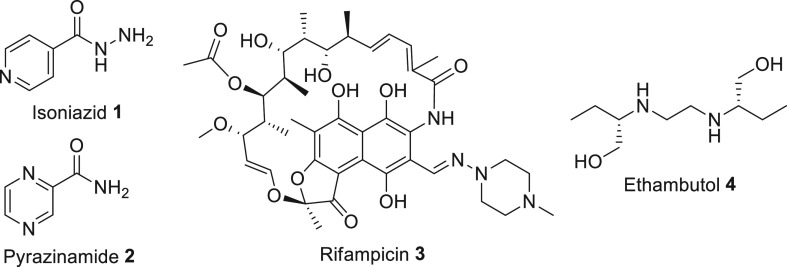
Structural representations of the front-line TB drugs.

Tuberculosis (TB) is an airborne illness caused by a single infectious agent, *Mycobacterium tuberculosis* (*Mtb*), continuing to be one of the world’s top ten infectious killers.^1^*Mtb* is predicted to infect around 2 billion people (mostly in the latent form), with a risk of individuals contracting the disease’s most aggressive form (generally 5–10% of the cases, predominantly among those with comorbidities such as diabetes or AIDS).^2^ Even though TB is usually treatable, it remains a worldwide concern. In 2020, over 1.3 million HIV-negative, together with 214000 HIV-positive individuals, died of TB. Worldwide, 9.9 million new cases of TB were reported in the same year, with men accounting for 56% of this total, 33% in adult women, and 11% in children.^2^ Additionally, the spread of multidrug-resistant (MDR) and extensively drug-resistant (XDR) tuberculosis and the simultaneous pandemic of HIV-TB coinfection, together with a deficient health care infrastructure and the lack of an effective vaccine, all contribute to the disease’s endurance.^3,4^ The current anti-*Mtb* pharmaceutical combination treatment, developed more than 40 years ago, consists of a four-drug regimen comprising isoniazid (**1**, INH), pyrazinamide (**2**, PYR), rifampicin (**3**, RFP) and ethambutol (**4**, EMB) (Figure 1).^4,5^ These therapies present disadvantages, including lengthy treatments, undesirable side effects, drug interactions, and poor patient compliance,^6,7^ in addition to the emergence of mycobacteria mutations conferring resistance to the drugs of this combination therapy. Consequently, the search for more effective drugs and therapy regimens has been critical in maintaining disease control.^8^

One strategy for treating tuberculosis has been to target the mycobacterial cell wall.^9^ The front-line TB drugs EMB and INH inhibit key enzymes involved in producing arabinogalactan and mycolic acids, which are noncovalently connected to a protein- and polysaccharide-based outer capsule.^10−13^ Numerous new drug families have been found by whole-cell screening that target essential proteins implicated in the construction of the cell wall components.^9^

**Figure 2:**
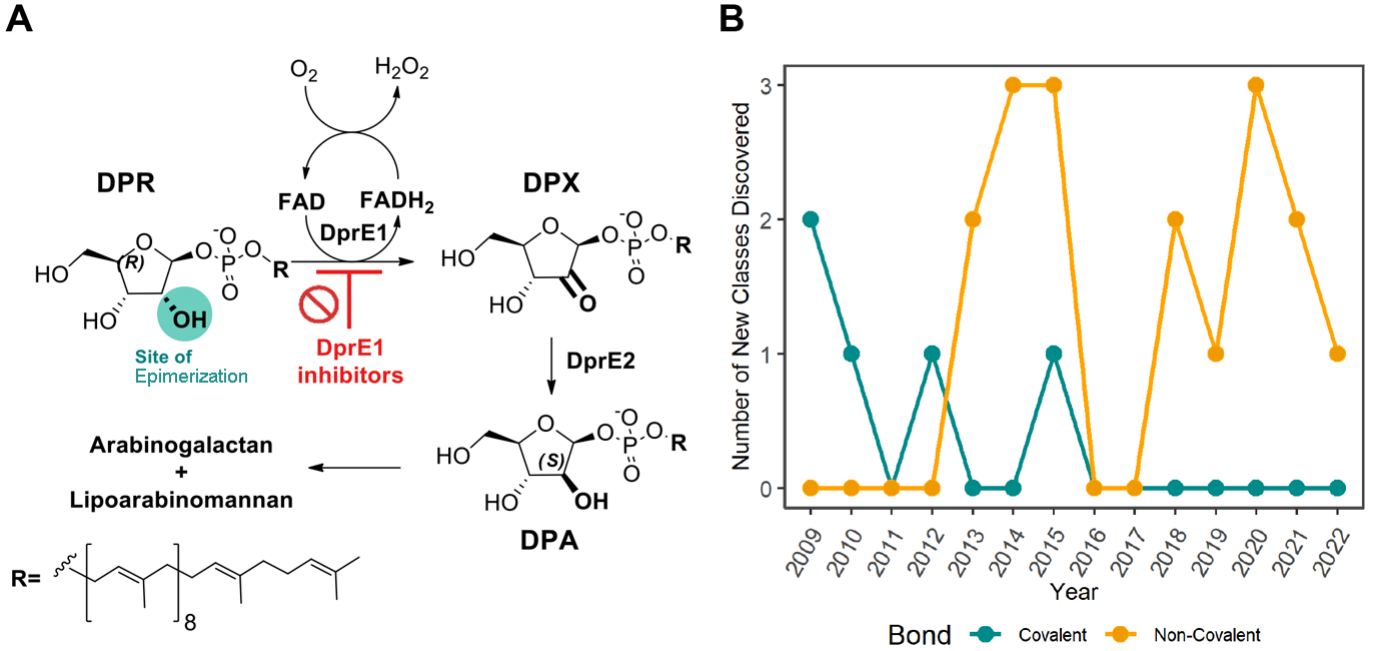
(A) The DPA biosynthetic pathway: in the presence of the cofactor FAD, the DprE1 and DprE2 enzymes catalyze the epimerization of the 2′-OH group in DPR to form DPA. (B) Timeline of the discovery of the different DprE1 classes.

DprE1, also known as decaprenylphosphoryl-β-d-ribose 2′-epimerase, is an indispensable flavoenzyme involved in forming the *Mtb* cell wall.^14^ It catalyzes the two-step epimerization of decaprenyl-phospho-ribose (DPR) to decaprenyl-phospho-arabinose (DPA), the precursor for arabinogalactan and lipoarabinomannan synthesis, in conjunction with decaprenylphosphoryl-d-2-keto erythro pentose reductase (DprE2, Figure 2-A).^14−16^ DprE1 initiates the first step of the epimerization process, where DPR is oxidized to the intermediate decaprenyl-phospho-2′-keto-d-arabinose (DPX), cofactored by flavin adenine dinucleotide (FAD), yielding FADH_2_. DprE2, which is NADH-dependent, subsequently converts DPX to DPA.^17−19^ The epimerization happens in the periplasmic region, which explains DprE1’s vulnerability as a target,^19^ making this flavoenzyme a promising target for developing novel therapeutic candidates to tackle TB. The druggable yet promiscuous nature of DprE1 has led to a significant number of DprE1 inhibitors with diverse molecular scaffolds and pharmacological profiles,^20−25^ as evidenced by an increasing number of publications on the subject. There have been 23 new classes of DprE1 inhibitors identified with antimycobacterial activity, and their different scaffolds are displayed in Tables 1 and 2. These inhibitors are divided into two types, according to their mechanism of action (MoA): (1) covalent binders, where five classes have been shown to irreversibly inhibit DprE1 by generating a covalent adduct with the C387 residue, and (2) noncovalent inhibitors, in which 17 reported classes were experimentally confirmed to act as competitive inhibitors (Figure 2B). Several DprE1 inhibitor reviews have been written during the past decade, covering both scaffold and docking studies.^20−24,26,27^

**Table 1:**
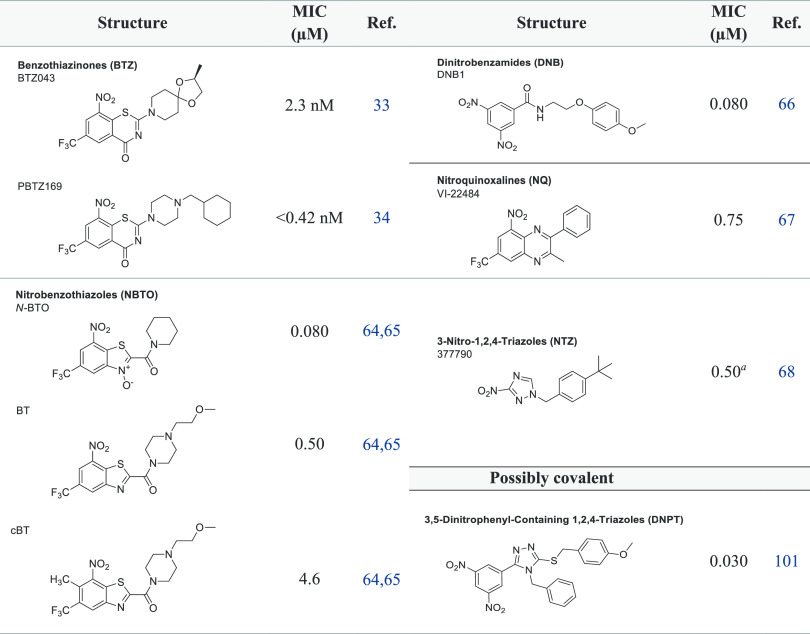
Reported Compounds Targeting DprE1 Covalently

a MIC_90_.

**Table 2:**
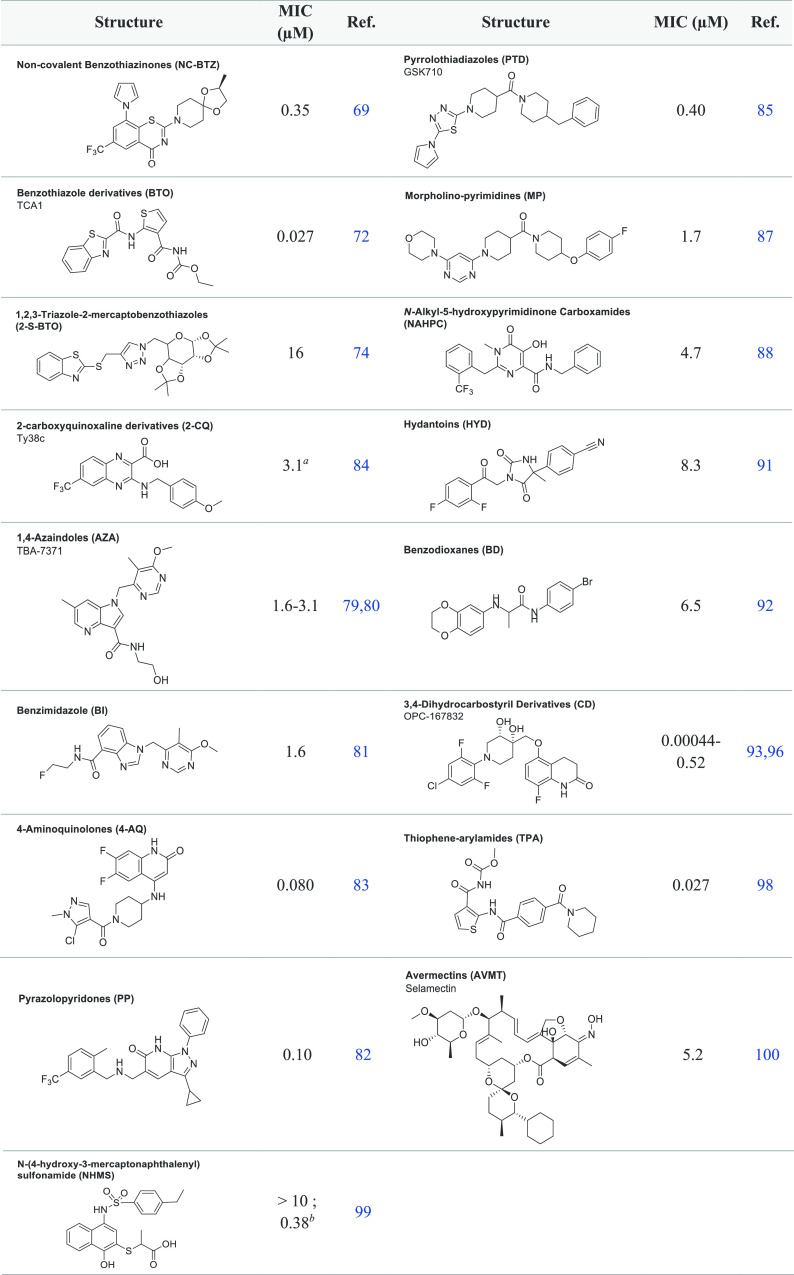
Reported Compounds Targeting DprE1 Noncovalently

a MIC_99_;

b MIC_50_*Ms*

### DprE1 Covalent Inhibitors

**Figure 3:**
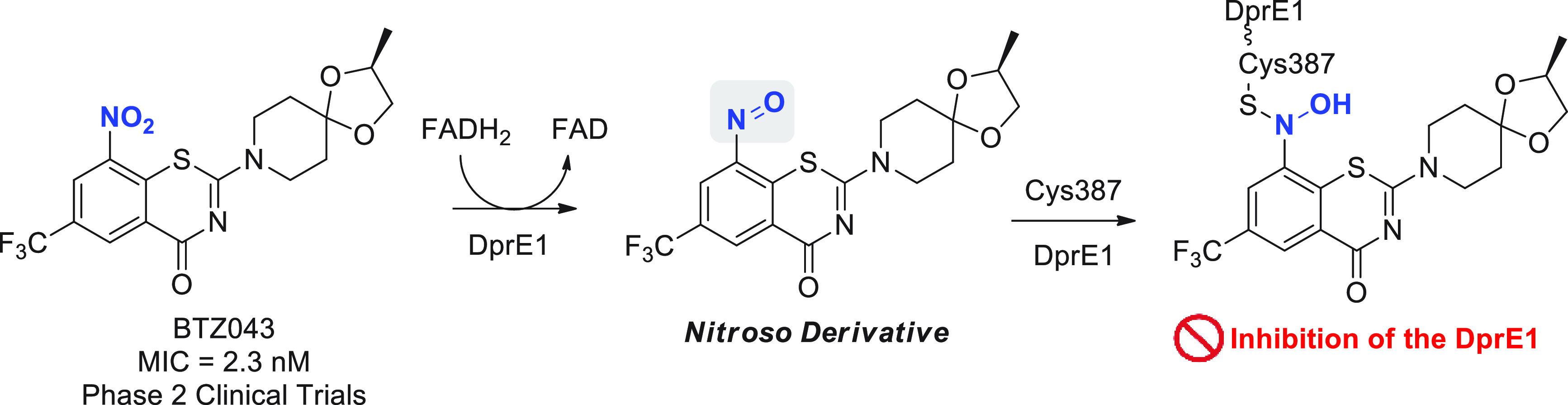
Bioactivation of BTZ043 by DprE1 and cofactor FADH_2_, and subsequent nucleophilic addition by Cys387.

DprE1 was first discovered as a target of benzothiazinones, which inhibit the flavoenzyme DprE1 irreversibly through the generation of a covalent adduct with the amino acid Cys387. A fundamental similarity of the covalent DprE1 inhibitors is the presence of a nitro group on the molecule, which is required for its inhibition mechanism.^28^ Makarov and colleagues were the first to demonstrate this type of inhibition, in which they proved that benzothiazinones (BTZ) were capable of strongly suppressing DprE1 activity *in vitro* and *in vivo*. Compound BTZ043 (Figure 3) was shown to act as a prodrug in the presence of FADH_2_, where the nitro group on the benzothiazinone core is reduced to its nitroso derivative. The reactive nitroso form reacts with the thiol group on the Cys387 residue in DprE1, producing a semimercaptal bond with the amino acid residue and a covalent adduct that acts as a suicide substrate, irreversibly inhibiting the enzyme (Figure 3).^29−35^

Since then, more than ∼600 new nitrobenzothiazinones (BTZ; Table 1) have been described, and nearly 90% of these molecules proved active against *Mtb* (MIC < 10 μM).^30−63^ DprE1 has also been identified as the target of nitrobenzothiazoles (NBTO),^64,65^ dinitrobenzamides (DNBs),^66^ nitroquinoxalines (NQs),^67^ and 3-nitro-1,2,4-triazoles (NTZs),^68^ all of which interact as covalent inhibitors (Table 1).

### DprE1 Noncovalent Inhibitors

Numerous scaffolds acting noncovalently also have been investigated for their activity against *Mtb* and are depicted in Table 2. These inhibitors include non-nitro BTZ analogues (NC BTZ),^69−71^ benzothiazoles (BTO),^72,73^ 1,2,3-triazole-2-mercaptobenzothiazoles (2-S-BTO),^74^ 1,4-azaindoles (AZA),^75−80^ benzimidazoles (BI),^81^ pyrazolopyridones (PP),^82^ 4-aminoquinolone piperidine amides (4-AQ),^83^ 2-carboxyquinoxaline derivatives (2-CQ),^84^ pyrrolothiadiazoles (PTD),^85,86^ morpholine-pyrimidines (MP),^87^*N*-alkyl-5-hydroxypyrimidinone carboxamides (NAHPC),^88,89^ hydantoins (HYD),^90,91^ benzodioxanes (BD),^92^ 3,4-dihydrocarbostyril derivatives (CD),^93−97^ thiophene-arylamide compounds (TPA),^98^*N*-(4-hydroxy-3-mercaptonaphthalenyl) sulfonamides (NHMS),^99^ and avermectins (AVMT).^100^

## Physicochemical and ADMET Properties of DprE1 Inhibitors

Numerous research groups have examined the connections between small molecules’ physicochemical (PC) descriptors, potency, and ADMET profile.^102−104^ PC descriptors can affect efficacy, safety, or metabolism. Numerous molecular descriptors have been shown to be useful in predicting ADMET characteristics and have been used to characterize a variety of molecular properties, including lipophilicity, molecular flexibility, hydrogen-bonding ability, and molecular weight.^103,105^ Additionally, small-molecule-based pharmacological candidates must be sufficiently permeable and soluble to allow experimental testing and have the capability to reach their site of action as well as to activate their main targets, for which the PC descriptors are critical.^106^ Research on the chemical space exploration of DprE1 inhibitors found a significant lipophilic character, establishing a different cluster from currently available tuberculosis medicines, as shown by principal component analysis from their physicochemical descriptor analysis.^107^ Thus, ongoing research is essential to gain new insights into the design and development of highly active covalent and noncovalent DprE1 inhibitors and guiding hit and lead optimization to produce nonhazardous small-molecule-based treatments against *Mtb*.

### Data Collection and Preprocessing

To investigate the molecular diversity and ADMET properties of the DprE1 inhibitors disclosed in this review, we collected a data set of a total of 1519 structurally diverse molecules by reviewing the literature from the year 2009 to April 2022.^28−101,108−119^

The data set was split by two subsets, covalent (Cov) and noncovalent (NCov) binders, and then each compound was classified as active (MIC < 10 μM, **Act**) or not active (MIC ≥ 10 μM, **NAct**), following the MIC cutoff criteria adapted by the report of Makarov et al.^26^ The PC descriptors molecular weight (MW), lipophilicity through calculated partition coefficient (*C* log *P*), distribution coefficient at pH = 7.4 (*C* log *D*), intrinsic aqueous solubility (log *S*), hydrogen bond acceptors and donors (HBAs and HBDs), topological polar surface area (TPSA), number of rotatable bonds (ROTBS), and flexibility index (FInd) were computed by StarDrop v7.2.0.32905.^120^ The median (Md), mean (Mn), standard deviation (SD), Student *t*-test analyses were implemented and analyzed. Drug design oriented rules such as Lipinski’s Rule of 5 (Ro5),^121^ GSK’s 4/400 rule,^122^ and Pfizer’s 3/75 rule^123^ were also explored in this work. The ADMET predictions, CYP inhibition and metabolism, blood–brain barrier (BBB) penetration, plasma protein binding (PPB), P-glycoprotein (P-gp) substrate classification, and pan-assay interference compound (PAINS) count were obtained with StarDrop v7.2.0.32905,^120,124−126^ and structure alerts were processed through ChemBioServer 2.0.^127^ The generated raw data were then analyzed using manual R scripts in RStudio (Version 1.4.1106). Prior to processing, any observation with missing values was removed using the na.omit function, and the graphic figures were produced using the ggplot2 package.

### QSAR Metrics to Evaluate Model Performance

When appropriate, an analysis of the predictive model was conducted in which numerous model performance metrics for a classification model were calculated. Internally, we used four measures: (1) accuracy (ACC), (2) precision, (3) sensitivity, and (4) specificity. The following equations show their corresponding definitions^128^
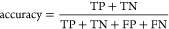
1
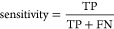
2
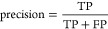
3
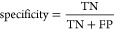
4where TP are true positives, TN are true negatives, FP are false positives, and FN are false negatives.^128^

## Correlations of MIC with IC_50_ DprE1 and with CC_50_

**Figure 4:**
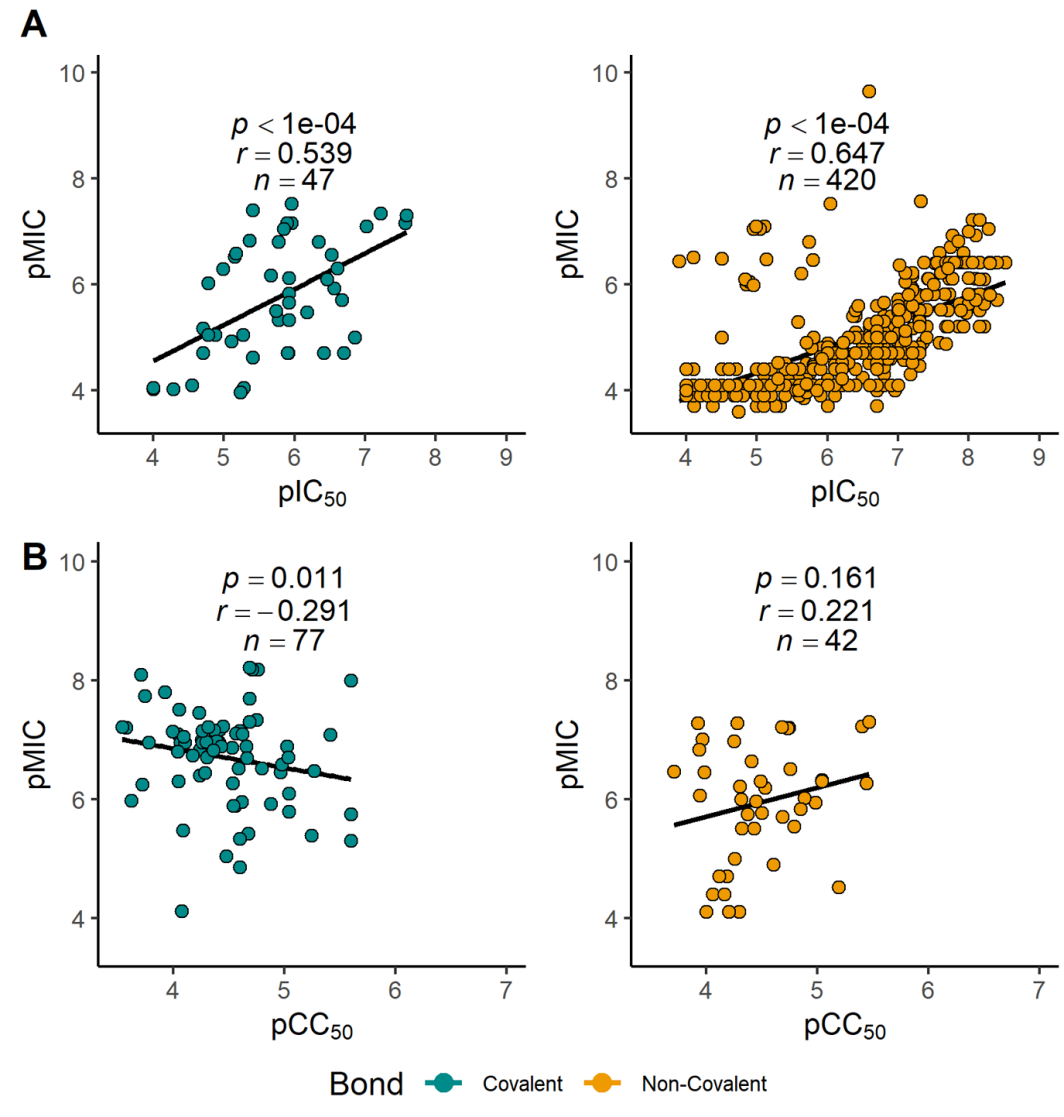
(A) Scatter plot of DprE1 pIC_50_ (−log_10_[IC_50_ (molar)]) versus pMIC (−log_10_[MIC (molar)]) against *Mtb* for covalent (left) and noncovalent (right) inhibitors and Pearson correlation coefficient between DprE1 pIC_50_ and pMIC. (B) Scatter plot of cytotoxicity pCC_50_ (−log_10_[CC_50_ (molar)]) versus pMIC for covalent (left) and noncovalent (right) inhibitors and Pearson correlation coefficient between pCC_50_ and pMIC.

To establish a correlation between the DprE1 enzyme inhibition and the subsequent MIC between the different classes of inhibitors, we performed a Pearson’s correlation (between the experimental pIC_50_ DprE1 and pMIC values), and the results are described in Figure 4A. In both types of binders, a moderate to strong positive correlation is observed between DprE1 pIC_50_ and *Mtb* pMIC, with statistical analyses of our results showing a significant correlation (*p* < 0.0001), whereas noncovalent inhibitors display a higher positive correlation coefficient (*n* = 420, *r* = 0.647) than the covalent binders (*n* = 47, *r* = 0.539). These results reveal conclusively that antimycobacterial efficacy significantly depends on the inhibition of the flavoenzyme DprE1. Several of these inhibitors were investigated for cytotoxicity in various human cell lines, including A549,^64,67,77,82,83^ HeLa,^37,39^ HepG2,^28,56,66,69,72,74,87,88,90−92,109,111^ J-774,^111^ THP-1,^60^ and Vero cell lines.^9,37−42,44,47−49,51,52,54,98,108,113,119^ A small negative correlation (*r* = −0.291, *p* = 0.011, Figure 4B) was found between the experimental cytotoxicity concentrations (CC_50_) and MIC, for covalent binders. This observation suggests that even the most effective covalent binders appear to display a safe profile, encouraging the ongoing search for novel inhibitors. In contrast, for the noncovalent binders, a Pearson’s correlation analysis did not reveal the existence of a correlation between pCC_50_ and pMIC (not statistically significant, *n* = 42, *r* = 0.221, *p* = 0.161, Figure 4B).

### The Impact of Nine Molecular Properties

**Figure 5:**
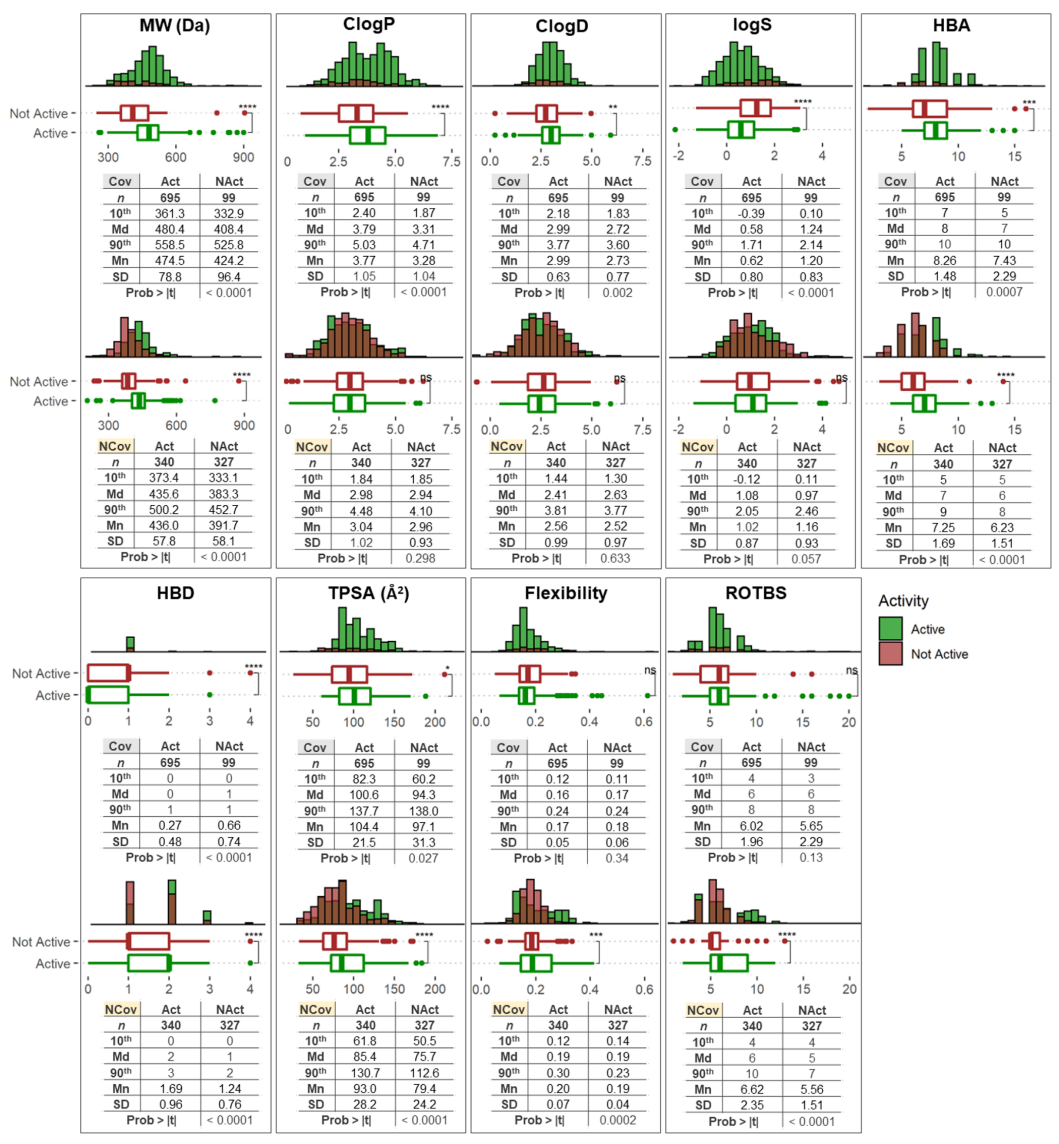
Physicochemical property distribution and statistics of the inhibitor (**Act**, in green) and noninhibitor (**NAct**, in red) classes. Each corresponding binding type (covalent (Cov) upper and noncovalent (NCov) above) is shown for MW, *C* log *P*, *C* log *D*, log *S*, HBA, HBD, TPSA, flexibility, and ROTBS. *N* indicates the total number of compounds considered in each analysis. A two-sided Student’s *t*-test was used to determine the statistical significance of active and inactive compounds, among those classified as covalent or noncovalent inhibitors, and the *p* values were evaluated (ns *p*-value >0.05, *p value <0.05, **p value <0.01, ***p value <0.001, ****p value <0.0001; Md, median; Mn, mean).

The nine molecular properties for the active (≤10 μM, **Act**) and nonactive classes (>10 μM, **Nact**), considering separately the covalent and noncovalent molecules, are represented in Figure 5. We evaluated the significance of the difference between the means by a two-sided Student’s *t*-test.

#### Molecular Weight

The molecular weight (MW) is a critical property in the development of small-molecule drugs.^129,130^ It has the potential to influence a variety of molecular processes, including absorption, blood–brain barrier (BBB) penetration, bile elimination rate, and interactions with biological targets, being frequently investigated as part of the process for compounds optimization.^131,132^

##### Covalent Inhibitors

The MW values for most of the drugs varied from 361.3 Da (10th percentile) to 558.5 Da (90th percentile), with a median MW value of 480.4 Da. Nonactive compounds generally had a lower molecular weight, with most molecules ranging from 332.9 Da (10th percentile) to 525.8 Da (90th percentile) and a median MW value of 408.4 Da. Comparison of the MW for active and nonactive candidates also showed that the set of actives had a higher mean MW (474.5 Da) than the set of nonactives (424.4 Da) by 50.1 Da, which proved statistically significant (*p* < 0.0001).

##### Noncovalent Inhibitors

The MW values for most of the compounds varied from 373.4 Da (10th percentile) to 500.2 Da (90th percentile), with a median MW value of 435.6 Da. Nonactive molecules had a lower molecular weight, with most of the molecules ranging from 333.1 Da (10th percentile) to 452.7 Da (90th percentile) and a median MW value of 383.3 Da. A comparison of the MW for active and nonactive candidates also showed that the set of active molecules had a higher mean MW (436.0 Da) than the set of nonactive molecules (391.7 Da) by 44.3 Da, which was statistically significant (*p* < 0.0001).

#### Lipophilicity

Lipophilicity, as indicated by the *C* log *P* and *C* log *D* values obtained here, is critical in defining key ADMET characteristics and potency. For instance, when lipophilicity levels are high, metabolism and solubility are more susceptible to being impaired, while low lipophilicity may increase permeability.^133^

##### Covalent Inhibitors

Covalent molecules in the active set displayed a similar *C* log *P* range, though right-shifted to higher lipophilicity, with 10th to 90th percentile values of 2.40 to 5.03 and a higher mean value of 3.77 versus 3.28 (*p* < 0.0001), compared to the nonactive counterparts (1.87 for the 10th percentile, 4.71 for the 90th percentile). Regarding the *C* log *D* values, the covalent binders in the active set showed a similar *C* log *D* range to the nonactive counterparts, with 10th to 90th percentile values of 2.18 to 3.77 and a slightly higher mean value of 2.99 versus 2.73 (*p* = 0.002), compared to the nonactive counterparts (1.83 for the 10th percentile, 3.60 for the 90th percentile).

##### Noncovalent Inhibitors

An opposite situation is observed in the noncovalent binders, although in this case there was no statistically significant difference in both *C* log *P* (*p* = 0.298) and *C* log *D* (*p* = 0.633) properties.

#### Intrinsic Aqueous Solubility (log *S*)

The intrinsic aqueous solubility (log *S*) of an ionizable molecule is defined as its concentration in saturated aqueous solution at a particular temperature.^134^

##### Covalent Inhibitors

A comparison of log *S* for active and nonactive inhibitors showed that the active set had a lower mean of log *S* (0.58) than the nonactive set (1.24, *p* < 0.0001).

##### Noncovalent Inhibitors

In contrast, an assessment of log *S* values for the noncovalent inhibitors was not significantly different between active and nonactive sets (*p* = 0.057).

#### Hydrogen Bond Acceptors and Donors

HBAs and HBDs are additional significant descriptors for drug discovery that relate to the polarity and permeability of compounds.^131,135^ For example, it was revealed that while the properties HBAs and MW have increased considerably over time, HBDs and lipophilicity have remained rather consistent.^136^ These data suggest that counting HBDs may be more significant for drug development than counting HBAs, in which a higher number of HBDs can lead to very poor solubility, permeability, and bioavailability.^137^

##### Covalent Inhibitors

The active covalent inhibitor set displayed more HBAs (from 7 (10th percentile) to 10 (90th percentile) with a median HBA value of 8), compared to the nonactive counterparts (from 5 (10th percentile) to 10 (90th percentile) with a median of 7). The higher mean value of 8.26 to the active compounds was shown to be statistically significant against the nonactive (*x̅* = 7.43, *p* = 0.0007). The covalent active and nonactive inhibitors had a minimal number of HBDs, with the median value being 0 for the active and 1 for the nonactive sets. Comparison of the HBDs for active and nonactive candidates also showed that the active set had a lower mean HBD (0.27) than the nonactive set (0.66, *p* < 0.0001). A more significant number of HBAs in the active set is likely attributable to the enthalpic aspect of the binding process, in which H-bonding plays a crucial role in aligning the molecule/warhead to facilitate interaction with the active nucleophile site. Regarding the effect of HBDs leading to molecules with poor solubility, permeability, and bioavailability, the observation of a reduced number of HBDs in active compounds can be explained as the avoidance of a self-reaction of the molecules with their covalent warhead and the corresponding hydrogen-bond donor (e.g., −OH/NH/SH groups).

##### Noncovalent Inhibitors

The noncovalent active set displayed higher values of HBAs, from 5 (10th percentile) to 9 (90th percentile) with a median HBA value of 7, compared to the nonactive set (5, 10th percentile; 8, 90th percentile; 6, median). The higher mean value of 7.25 for the active compounds was shown to be statistically significant against the nonactive (*x̅* = 6.23, *p* < 0.0001). Unlike the case for the covalent binders, the noncovalent inhibitors showed a higher number of HBDs, with a median value of 2 for the active set and 1 for the nonactive. A comparison of the HBD for active and nonactive sets also showed that the active set had a higher mean HBD (*x̅* = 1.69) than the nonactive set (*x̅* = 1.24, *p* < 0.0001). This result is expected, given that the H-bonding potential via HBD or HBA would be greater in noncovalent analogues for active versus inactive. The analysis shows that increasing HBA/HBD for noncovalent inhibitors can be a strategy to increase potency by increasing a stronger binding via H-bonding on the binding site rather than increasing lipophilicity (*C* log *P* values were found to be not statistically significant in the noncovalent set).

#### Topological Surface Area

The topological surface area (TPSA) is another descriptor of importance in permeability and oral bioavailability estimates connected to hydrogen bonding (N and O atom count).^138^

##### Covalent Inhibitors

TPSA values ranged from about 82.3 Å^2^ (10th percentile) to 137.7 Å^2^ (90th percentile), with a median value of 100.6 Å^2^ for the actives set. Nonactives are left-shifted to a lower value of TPSA, with values varying from 60.2 Å^2^ (10th percentile) to 138.0 Å^2^ (90th percentile) and a median TPSA value of 94.3 Å^2^. A comparison of the TPSA for active and nonactive candidates also showed a higher mean TPSA value of 104.4 Å^2^ for the active set, compared to the nonactive set (97.1 Å^2^, *p* = 0.027).

##### Noncovalent Inhibitors

TPSA values varied from about 61.8 Å^2^ (10th percentile) to 130.7 Å^2^ (90th percentile) with a median value of 85.4 Å^2^ for the active set. Nonactives are left-shifted to a lower value of TPSA, with values varying from 50.5 Å^2^ (10th percentile) to 112.6 Å^2^ (90th percentile), with a median TPSA value of 79.4 Å^2^. Comparison of the TPSA for active and nonactive candidates also showed that the active set had a higher mean TPSA value (93.0 Å^2^) than the nonactive set (79.4 Å^2^, *p* < 0.0001). Noncovalent binders have lower TPSA values than covalent inhibitors, both active and inactive. This result is likely due to the existing electrophilic warhead in the covalent binders (acrylamide or nitro), which increases this PC descriptor. Similarly demonstrated with HBAs and HBDs, we can observe the H-bonding role in affecting the potency of the different types of inhibitors.

#### Flexibility Index

The flexibility index (FInd) is described as the ratio of rotatable bonds to total bonds. No statistically significant difference was observed between the active and nonactive sets, for the covalent inhibitors (*p* = 0.34). The noncovalent active set displayed values of FInd from 0.12 (10th percentile) to 0.30 (90th percentile) with a median FInd value of 0.19, compared to the nonactive set (0.14, 10th percentile; 0.23, 90th percentile; 0.19, median). The higher mean value of 0.20 for the active compounds was shown to be statistically significant against the nonactives (*x̅* = 0.19, *p* = 0.0002).

#### Number of Rotatable Bonds

Similarly, ROTBS for the covalent inhibitors were not significantly different between active and nonactive sets (*p* = 0.13), even though the means were quite similar between inhibitors (*x̅* = 6.02) and noninhibitors (*x̅* = 5.65). In contrast, for the noncovalent inhibitors the means for the ROTBS were statistically significant (*p* < 0.0001), with values of 6.62 for the active vs 5.56 for the nonactive sets. ROTBS values for most of the inhibitor set varied from 4 (10th percentile) to 10 (90th percentile) with a median ROTBS value of 6.

The analysis described above indicates that, for inactive covalent DprE1 inhibitors, it may be necessary to optimize the compounds by increasing MW, *C* log *P*, *C* log *D*, HBA, and TPSA while reducing log *S* and HBD, to match more closely the active set’s corresponding properties. Concerning reducing HBD, the presence of a hydrogen bond donor in a core with a chemically reactive warhead could lead to drug instability through self-reactivity (though less likely for *in situ* bioreductively activated warheads such as nitro heterocycles); therefore, this needs to be considered in line with the analysis. The inactive noncovalent DprE1 inhibitors indicate that compound optimization may benefit from increasing MW, HBA, HBD, TPSA, FInd, and ROTBS. This step change in properties will drive the enthalpic component of binding by enhancement of hydrogen bonding and enhancing the ligand conformation for optimal fit.

### Impact of Physicochemical Properties of DprE1 inhibitors on Oral Absorption

**Figure 6:**
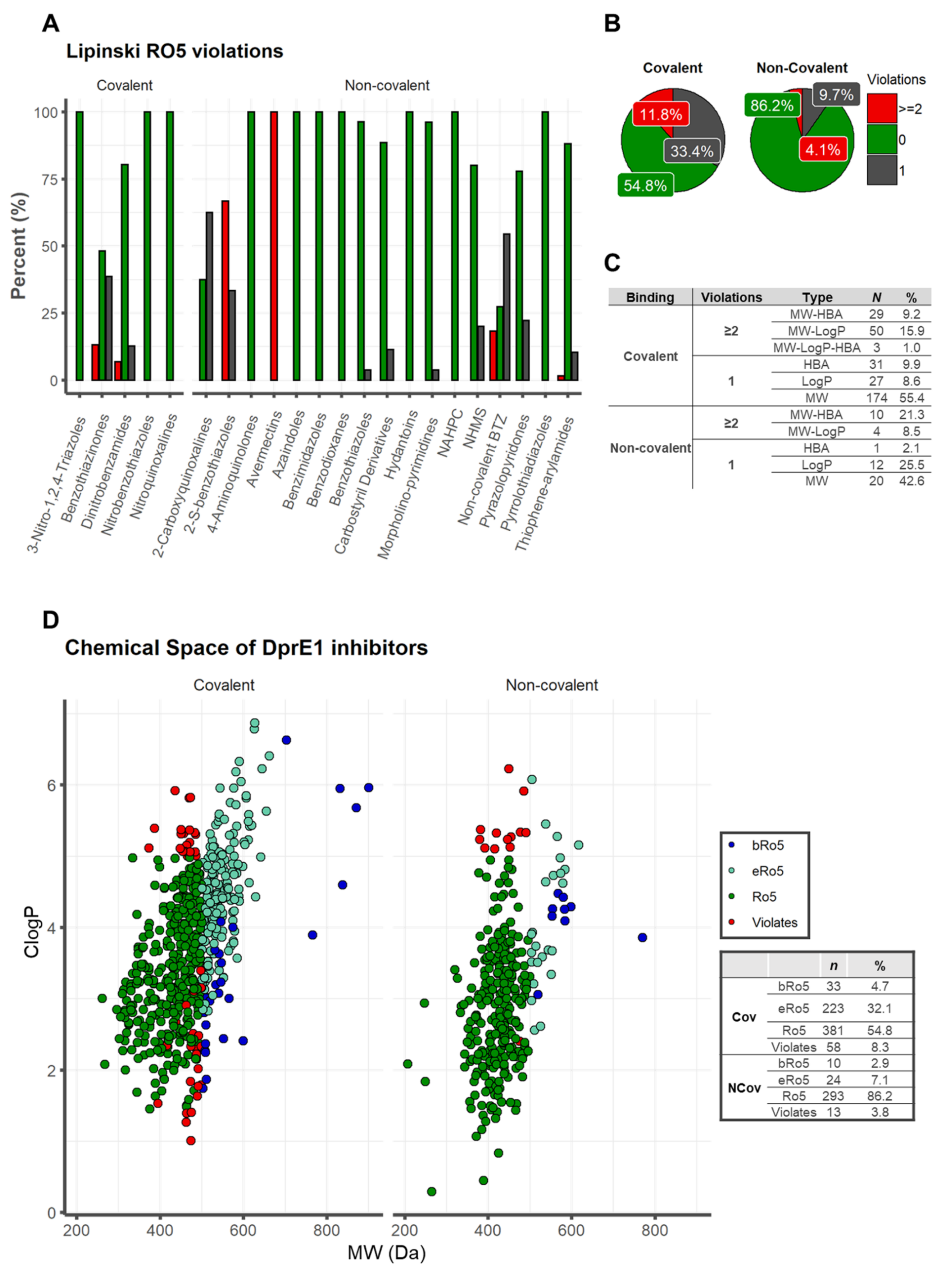
Lipinski’s Rule of 5 (Ro5): distribution of the number of Lipinski Ro5 violations for (A) each class of DprE1 inhibitors and (B) for the covalent and noncovalent category binders. (C) Ro5 violation types. (D) Physicochemical property space of covalent and noncovalent DprE1 inhibitors, with *C* log *P* as a function of MW.

Lipinski’s Rule of 5 (Ro5) indicates that if a molecule meets the criteria log *P* ≤ 5, MW ≤ 500 Da, HBAs (O + N atom count) ≤ 10 and HBDs (OH + NH count) ≤ 5, the compound is more likely to have membrane permeability and hence be more readily absorbed in the human digestive system via passive diffusion.^121,139^ These set limits were chosen to cover around 90% of the range for the four estimated PC descriptors, and the Ro5 is compromised when two or more criteria are exceeded.^121,139^ Our analysis reveals that 65.1% of active DprE1 inhibitors (*n* = 674/1035, MIC < 10 μM) do not show violations of the Ro5. Among the covalent binders, only 54.8% (381/695) fall inside the chemical space of Ro5, while a larger proportion is seen among the noncovalent binders [86.2% (293/340)] (Figure 6B). Within the covalent data set, NBTO, NQ, and NTZ score 100% for no Ro5 violations, while DNB and BTZ score 80.4% and 48.2%, respectively. The noncovalent data set proved more diverse, with 4-AQ, AZA, BI, BD, HYD, NAHPC, and PTD showing no violations (100%), and BTO (96.3%) > MP (96.2%) > CD (88.6%) > TPA (88.1%) > NMDS (80.0%) > PP (77.8%). 2-CQ and NC BTZ were the classes with a lower score of no violation (37.5, 27.3%) (Figure 6A). If we analyze both binding subsets which scored with one violation (25.6%, 265/1035), among covalent (33.4%, 232/695) and noncovalent (9.7%, 33/340) inhibitors, the classes of the covalent category were BTZ (38.6%) > DNB (12.8%) and those of the noncovalent were 2-CQ (62.5%) > NC BTZ (54.5%) > 2-S-BTO (33.3%) > PP (22.2%) > NMDS (20%) > CD (11.4%) > TPA (10.4%) > MP (3.8%) and BTO (3.7%) (Figure 6A). The main descriptor involved in Ro5 violations is MW, with a prevalence of 55.4% for the covalent and 42.6% for the noncovalent binders (Figure 6C). While analyzing the two subsets that scored at least two violations, we obtained for the covalent 11.8% (82/695) and for noncovalent 4.1% (14/340) (Figure 6B). The classes for the covalent inhibitors were BTZ (13.2%) > DNB (6.9%) and for the noncovalent counterparts AVMT (100%) > 2-S-BTO (66.7%) > NC BTZ (18.2%) and TPA (1.5%), respectively (Figure 6A). The most frequently used pair of PC descriptors in two Ro5 violations is MW–*C* log *P* for the covalent binders, with a frequency of 15.9%, and MW–HBA for noncovalent binders, 21.3%. The set MW-ClogP-HBA was found to be the most frequently violated for the compounds with three violations, with a score of 1.0% for the covalent binders (Figure 6-C). This finding was consistent with our PC descriptor analysis; nevertheless, covalent inhibitors exhibit higher molecular weight values and are more lipophilic than noncovalent binders. This property may impair oral bioavailability and should be considered during drug optimization.

### Mapping bRo5 and eRo5 Space

Lipinski’s rules delineate chemical space with compounds that “are more likely to be orally absorbed” as one way to describe chemical space, whereas “possible to be orally absorbed” is regarding the extended Ro5 (eRo5) and beyond Ro5 (bRo5). Understanding the precise boundaries of this chemical space will increase the likelihood of creating cell-permeable and orally accessible ligands for more difficult targets.^131,140,141^ Of the 1035 active compounds in this study, a large proportion (23.9%, *n* = 247/1035) cluster into what can be considered as an extension of Ro5 space (0 ≤ log *P* ≤ 7.5; 500 Da < MW ≤ 700 Da; HBDs (OH + NH count) ≤ 5; TPSA ≤ 200 Å^2^; ROTBS ≤ 20) and a natural tail of the distribution of compounds is based on Ro5 properties. Among these, 32.1% (223/695) are from the covalent class while only 7.1% (24/340) are from the noncovalent class. For the covalent and noncovalent inhibitors, only 4.7% (33/695) and 2.9% (10/340), respectively, were observed in oral bRo5 space (0 < log *P* or >7.5; 700 Da < MW ≤ 3000 Da; HBDs (OH + NH count) > 5; TPSA > 200 Å^2^; ROTBS > 20). A small proportion of DprE1 inhibitors, 6.9% (71/1035), did not fall into any Ro5 chemical space, with the highest proportion, 8.3% (58/695), for covalent and 3.8% (13/340) for the noncovalent binders (Figure 6-D).

### Distribution

The term “drug distribution” refers to how a substance is distributed across the body’s compartments. Certain factors, such as penetration through the central nervous system (CNS) or BBB, P-gp efflux, and PPB, can be adequately studied *in silico*. Additionally, since only the unbound (free) drug can interact with the target protein, the interaction of the drug with plasma proteins must be evaluated throughout the drug development process.^142^

#### Central Nervous System Penetration

**Figure 7:**
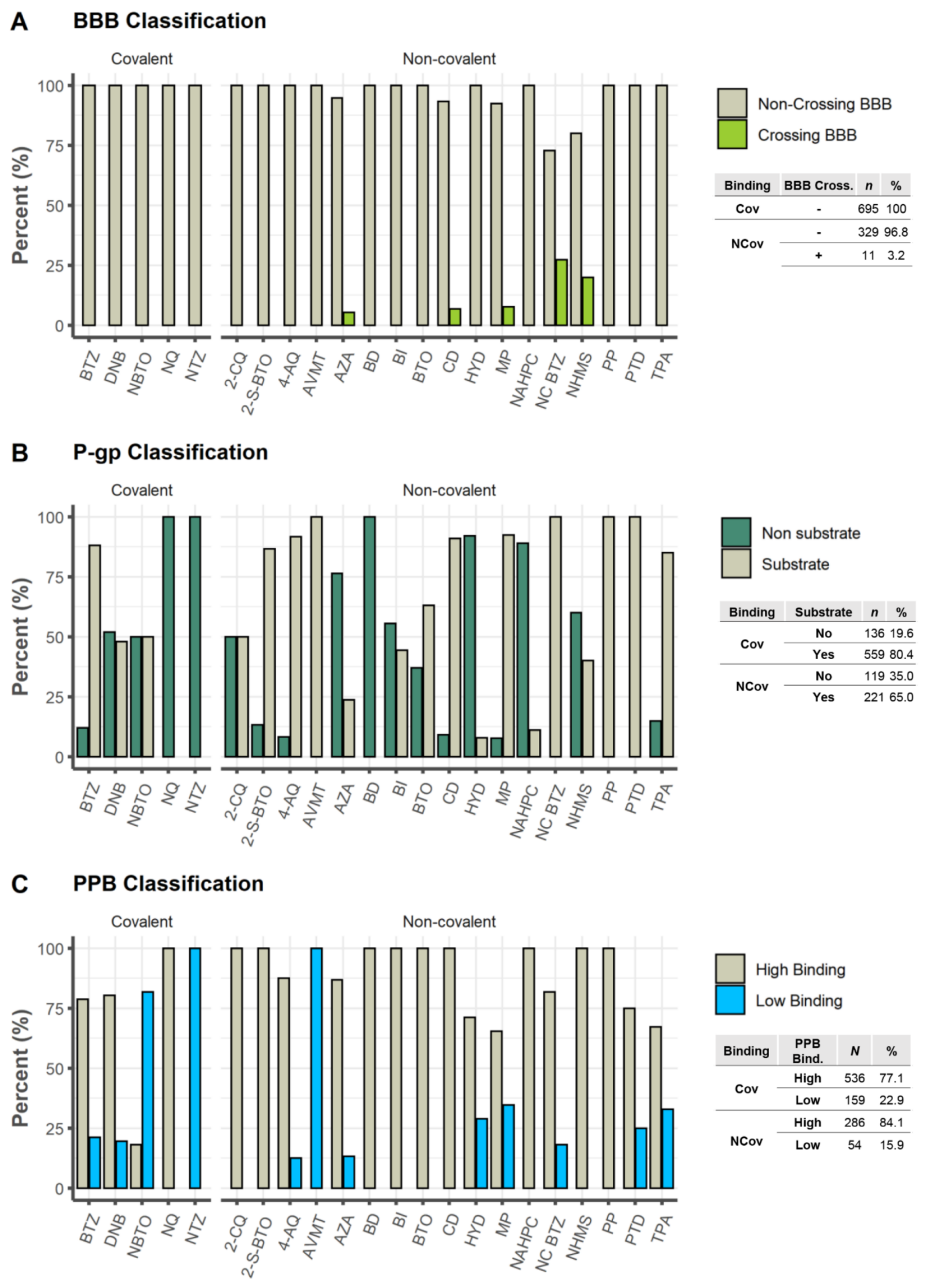
Analysis of some properties related to distribution: (A) Blood–brain barrier (BBB) penetration classification; (B) P-gp inhibitor classification; (C) plasma protein binding (PPB) classification.

For therapeutic CNS targets, good penetration is an essential requirement, but for non-CNS targets, the BBB penetration rate should be minimized to reduce potential neurotoxicity or adverse pharmacological events.^143^ StarDrop software uses the random forest classification model to classify if a molecule is a crossing or noncrossing of the BBB, while being one that employs descriptors compatible with the common fact that neutral molecules tend to penetrate the CNS more effectively than charged compounds and that cations normally permeate the CNS more effectively than anions.^120,124,125^ The predictive accuracy of BBB+ ranges from 80% to 100%, while that of BBB– ranges from 65% to 87%.^120,124,125^ Close to ∼99% of the total DprE1 inhibitors were predicted not to penetrate the CNS, with 100% within the covalent set and 96.8% for the noncovalent binders. Only 3.2% of the noncovalent inhibitors were found to have some BBB penetration, respectively NC BTZ 27.3% (3/11) > NHMS 20% (1/5) > MP 7.7% (2/26) > CD 6.8% (3/44) > AZA 5.3% (2/38) (Figure 7A).

#### P-gp Efflux System

P-gp is one of the most widely studied drug transporters to date, given the evidence of its presence in the majority of cells, including those of the intestinal mucosa and the BBB.^122^ We used the statistical model built-in to StarDrop v7.2.0.32905 to predict which DprE1 inhibitors could behave as P-gp substrates. It employs a random forest classification approach to classify compounds as probable or unlikely to be P-gp substrates. The model’s performance was evaluated on an independent test set of 51 chemicals, with 82% of nonsubstrates and 79% of substrates accurately categorized.^124^ A higher frequency of P-gp binders is predicted among the covalent inhibitors (80.4%), compared to the noncovalent class (65%). For the covalent inhibitor set, predictions point to P-gp substrates among the BTZ (88%) > NBTO (50%) > DNB (48%) and no P-gp substrates for the NTZ, NQ data sets (0%). For the noncovalent inhibitors set, predictions identify P-gp substrates among the AVMT, NC BTZ, PP, PTD (100%) > MP (92.3%) > 4-AQ (91.7%) > CD (90.9%) > 2-S-BTO (86.7%) > TPA (85.1%) > BTO (63%) > 2-CQ (50%) > BI (44.4%) > NHMS (40%) > AZA (23.7%) > NAHPC (11.1%) > HYD (7.9%) and no P-gp substrates for the BD (0%) (Figure 7B).

#### Plasma Protein Binding

The extent to which a drug binds to plasma proteins substantially affects its pharmacokinetic and pharmacodynamic effects. The drug’s efficacy will be proportional to the quantity of unbound drug in plasma. Additionally, the bound drug in plasma can operate as a reservoir for free drug clearance via various elimination pathways, lengthening the duration of action.^144,145^ From a QSAR model integrated into StarDrop v7.2.0.32905, Figure 7C categorizes and forecasts human PPB% (Hu PPB%) values for both covalent and noncovalent data sets. The model is a random forest that classifies the extent of plasma protein binding of test set substances as either “high” or “low” about the threshold above. Low-binding molecules are those that are less than 90% bound, and high-binding molecules are those that are more than 90% bound.^120,124,125^ It can be observed that both types of inhibitors display high binding capacity, with the noncovalent inhibitors scoring around 84.1% while the covalent inhibitors scored around 77.1%. For the covalent set, all inhibitors belonging to the NQ class displayed a high binding capacity (100%), this high tendency holding for DNB and BTZ classes (80.4% and 78.8%), while NBTO scores only around 18.2% and NTZ is predicted to have an irrelevant protein binding capacity (0%). For the noncovalent inhibitors, nine classes (2-CQ, 2-S-BTO, BD, BTO, BI, NAHPC, CD, NHMS, PP) were predicted to have a high binding capacity (100%), followed by 4-AQ (87.5%) > AZA (86.8%) > NC BTZ (81.8%) > PTD (75%) > HYD (71.1%) > TPA (67.2%) > MP (65.4%). AVMT is predicted to have an irrelevant protein binding capacity.

A comparison of the experimental PPB% values of the covalent and noncovalent binders to the StarDrop model results was performed, and the corresponding data sets are depicted in Tables 3 and 4. Regarding the covalent inhibitors, the literature examination returned a total of 21 compounds, including 16 BTZ^44,146^ and 5 NBTO^64^ molecules. The compounds with experimental data were employed to evaluate the performance metrics of the classification model from StarDrop, and the results and confusion matrix are displayed in Table 3. The obtained accuracy (ACC) rating of 81% indicates that the classification of ∼8 of every 10 molecules is correct. According to the StarDrop manual, the accuracy of the Plasma Protein Binding Classification (90%) model is 81%, which is consistent with the obtained predictions using covalent inhibitors. The precision value of 100% indicates that all the molecules predicted with high PPB% were correctly identified, and a sensitivity value of 80% reveals that 20% of the molecules with high PPB% were lost during the model application. The specificity value of 100% indicates that all molecules with low PPB% were accurately labeled.

**Table 3:**
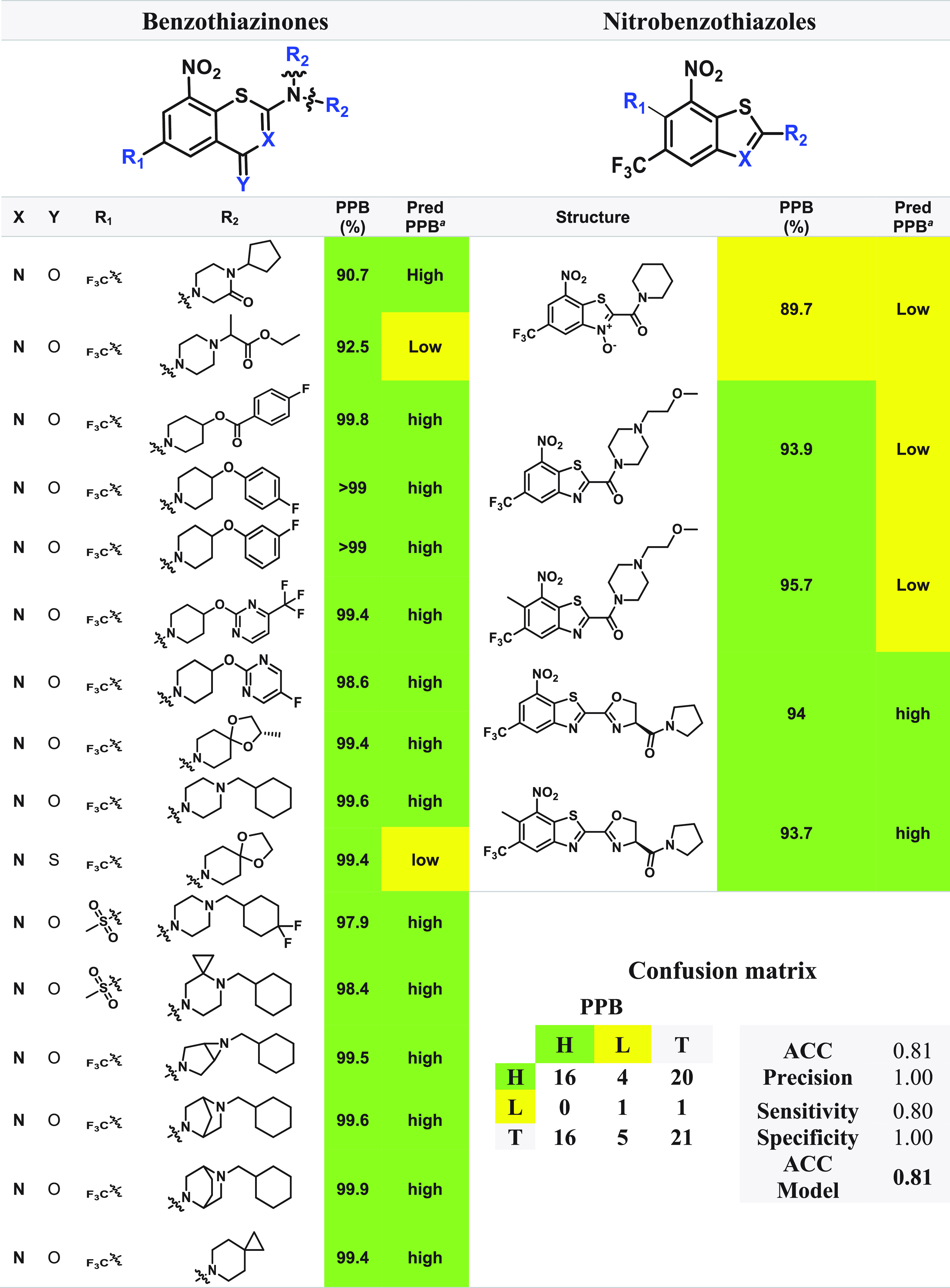
Experimental and Corresponding Predicted Plasma Protein Binding (PPB) Data, Confusion Matrix, and Performance Metrics Evaluating the PPB Data for Covalent Inhibitors

a Pred PPB: predicted plasma protein binding computed by StarDrop v7.2.0.32905. Legend: H, high (in green); L, low (in yellow).

**Table 4:**
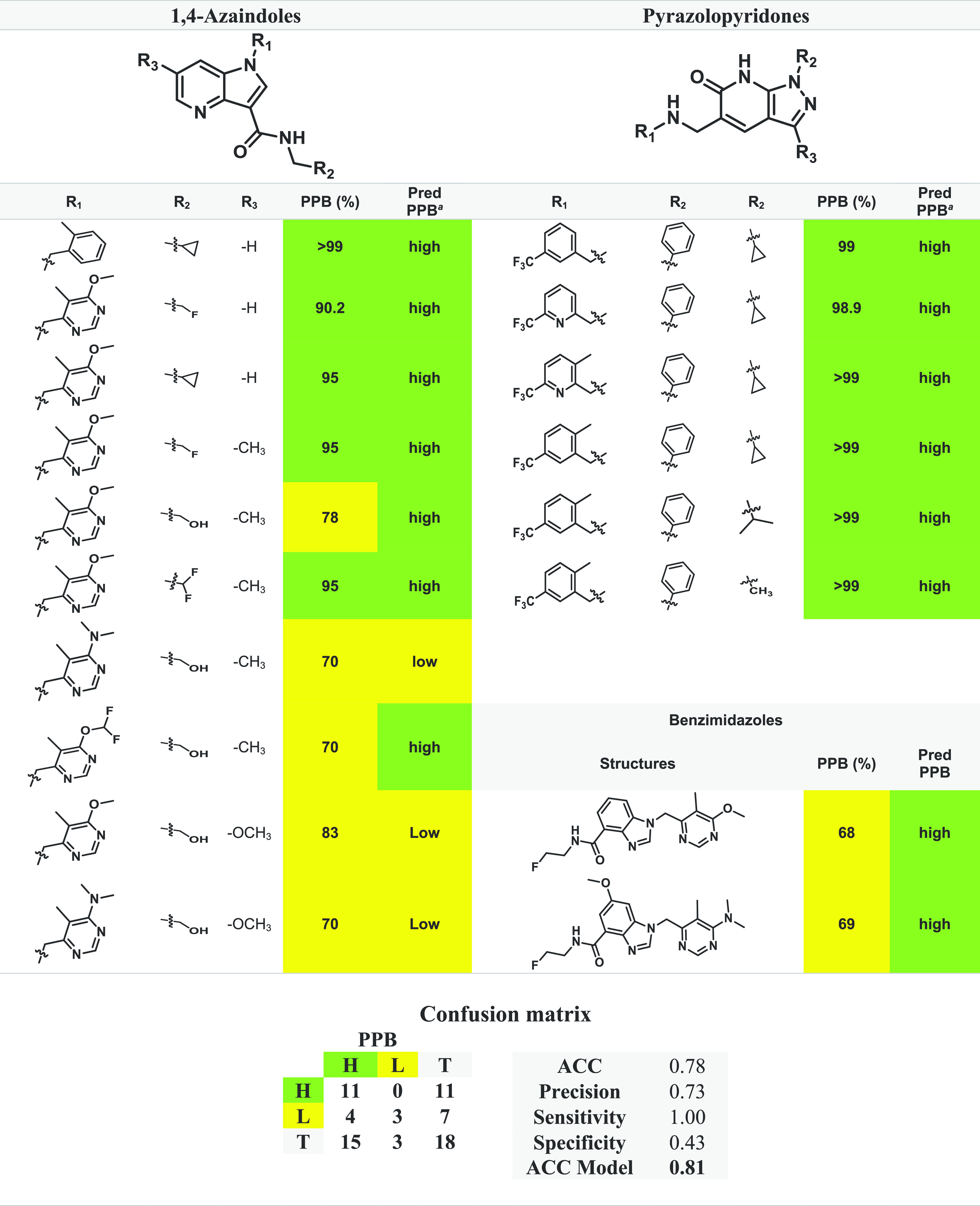
Experimental and Corresponding Predicted PPB data, Confusion Matrix, and Performance Metrics Evaluating the PPB Data for Noncovalent Inhibitors

a Pred PPB: predicted plasma protein binding computed by StarDrop v7.2.0.32905. Legend: H, high (in green); L, low (in yellow).

For the noncovalent inhibitors, the literature analysis provided a total of 18 compounds, including 10 AZA,^78,79^ 6 PP,^82^ and 2 BI^81^ molecules. These compounds with experimental data were also employed to evaluate the performance metrics of the classification model from StarDrop, the results and confusion matrix being displayed in Table 4. The obtained accuracy (ACC) rating of 78% indicates that classification of ∼8 of every 10 molecules is correct, with a similar accuracy value being reported from StarDrop’s manual (81%). The precision value (73%) indicates that 73% of the molecules predicted with high PPB% were correctly identified, and a sensitivity value of 100% reveals that no false negative value was predicted. The specificity value of 43% indicates that 57% of the molecules with low PPB% were mislabeled as high PPB% (false positive).

High plasma protein binding restricts the distribution of xenobiotics from the blood to tissues, affecting their metabolism, also holding a significant role in drug–drug interactions. Therefore, a reasonable predictive model with high sensitivity is required to avoid losing high PPB molecules during prediction and a high precision to prevent excess false positive results. Both model analyses for the experimental data of each class were shown to have a high sensitivity (Cov = 0.80 and Ncov = 1) together with high precision (Cov = 1 and Ncov = 0.73). Even though the sample size is relatively small in both testing sets (*N*(Cov) = 21 and *N*(Ncov) = 18), this study using experimental data reveals that the StarDrop model can reasonably predict plasma protein binding for both covalent and noncovalent binders and can be fairly reliable to assist with the development of DprE1 inhibitors.

To summarize this section, our computed analyses, along with literature values, indicate that both covalent and noncovalent DprE1 inhibitors are projected to be nonpermeable to the blood–brain barrier and to have a moderate to high plasma protein binding affinity. Covalent DprE1 inhibitors are more likely to be possible substrates (80.4%) for P-gp substrate transporters than their noncovalent counterparts (65%).

### Cytochromes P450 Metabolism

**Figure 8:**
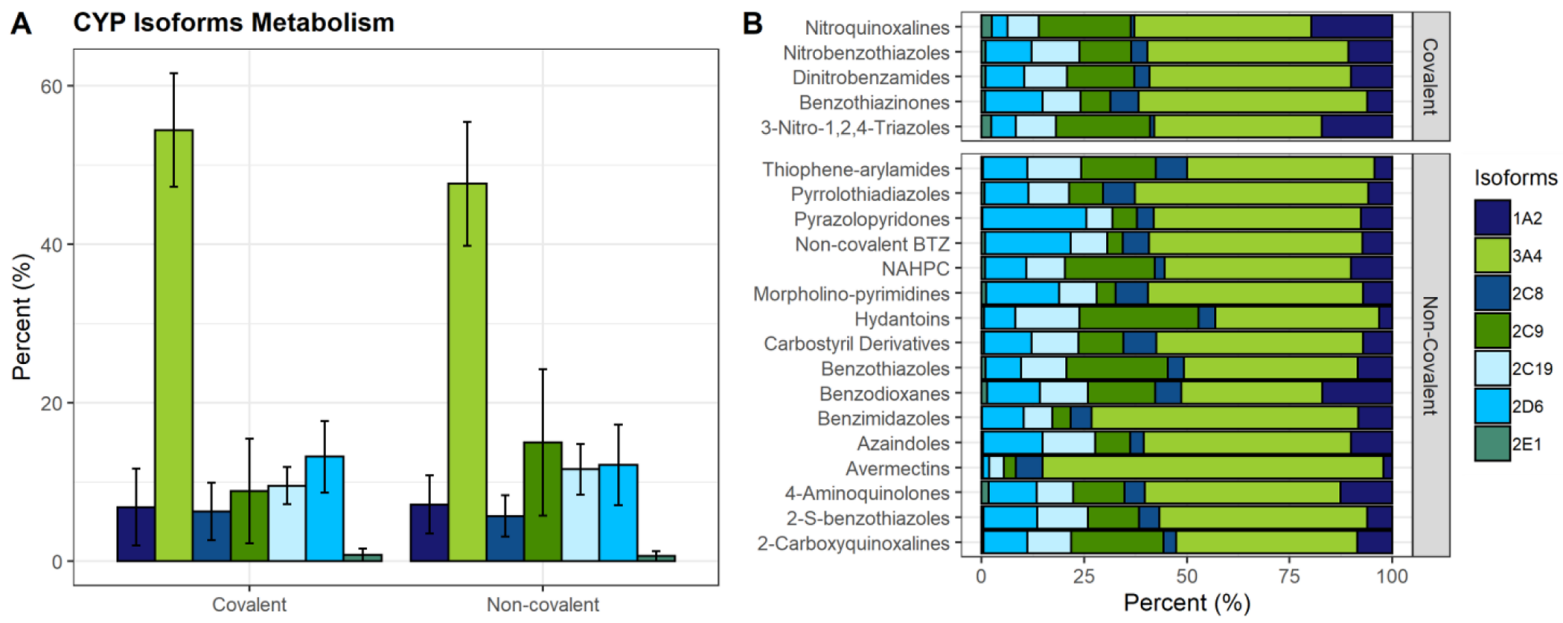
CYP isoform metabolism for (A) all covalent and noncovalent inhibitors and (B) each corresponding class of the covalent and noncovalent inhibitors.

Six cytochromes P450 (s) alleles are particularly important in drug metabolism: CYP1A2, CYP2C9, CYP2C19, CYP2D6, CYP2E1, and CYP3A4. They catalyze the oxidative metabolism of about 90% of human drugs and are the main determinants of the systemic clearance and bioavailability of these molecules.^147,148^ To evaluate which compounds in our data set might be CYP binders, we used the StarDrop WhichP450 module. Predictions of CYP isoform metabolism for each class of inhibitors are displayed in Figure 8. Calculations were conducted to determine the drug’s mean probability of being metabolized by related isoforms, indicating that it could be a candidate substrate. The computed predictions suggest that the DprE1 inhibitors would be metabolized mainly by the 3A4 isoform, with mean values of 54.4% and 47.6% of metabolism prediction for covalent and noncovalent binders, followed by the isoforms 2D6, 2C19, 2C9 (13.21%, 9.53%, and 8.86%) for the covalent inhibitors and 2C9, 2D6, 2C19 (15.09%, 12.16%, and 11.61%) for the noncovalent inhibitors. This set of molecules reveals a low proportion of metabolism from the 1A2, 2C8, and 2E1 isoforms (6.84%, 6.28%, and 0.83% for covalent inhibitors and 7.16%, 5.72%, and 0.66% for noncovalent inhibitors; Figure 8A). Regarding the corresponding moiety class for each isoform, AVMT was found to be the class with highest prediction to be metabolized by 3A4 isoform (83.0%) and BD the lowest (34.3%); NQ scored the highest prediction to be metabolized by the 1A2 isoform (19.67%) and AVMT the lowest (2.2%). CD showed the highest metabolism prediction (8.03%) for the 2C8 isoform and NQ the lowest (0.93%). HYD was the class with the highest probability to be metabolized by the 2C9 isoform (28.9%) and AVMT the lowest (3.0%). Regarding the 2C19 isoform, the HYD class was predicted to have the highest probability (15.7%) and AVMT the lowest (3.6%). For the 2D6 isoform, PP displayed the highest value (25.3%) and AVMT the lowest (1.4%). For the 2E1 isoform, NQ had the highest metabolism prediction (2.53%) and BI the lowest (0.13%, Figure 8B). These findings should be interpreted cautiously, as building appropriate prediction models is challenging due to the complicated chemical mechanisms underlying CYP metabolism,^142^ but they allow for a broad comparison of the various classes of compounds. These predictions contribute to our understanding of the role of the CYP superfamily in the metabolic stability of DprE1 inhibitors.

### Safety Profile

**Figure 9:**
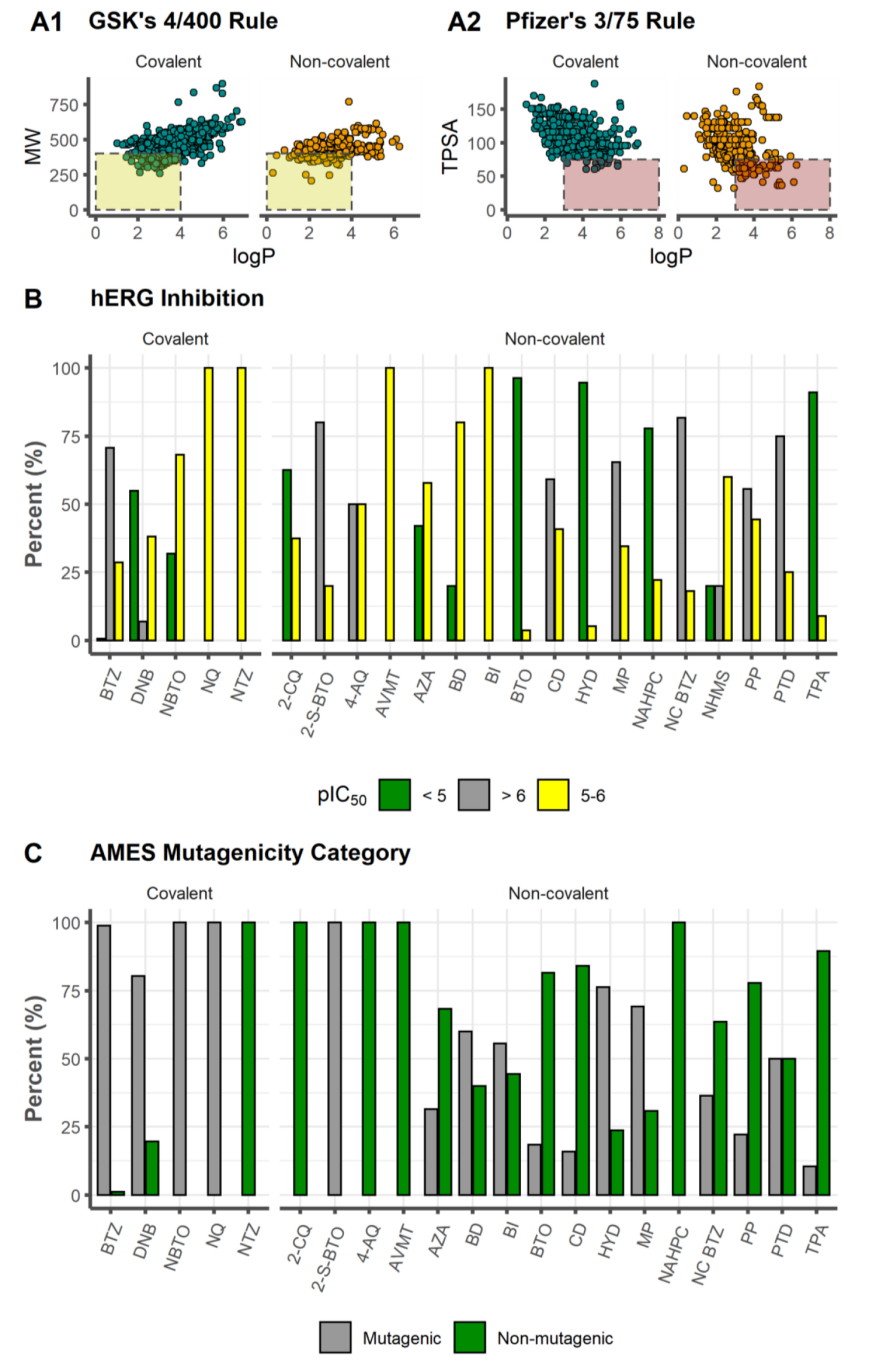
(A1) MW as a function of *C* log *P*. The yellow area indicates conformity by GSK’s 4/400 Rule. (A2) TPSA as a function of *C* log *P*. The red area indicates the molecules that failed on Pfizer’s 3/75 Rule. (B) hERG inhibition. (C) AMES mutagenicity category.

In addition to bioavailability, the safety profile is important, since it details the harmful consequences associated with the chemical substances under study. The analysis of log *P* versus MW, GSK’s 4/400 rule (log *P* ≤ 4 and MW ≤ 400 Da)^122^ for the evaluation of ADMET liabilities, shows that 17.1% (*n* = 177/1035) of the active inhibitors fall in the more desirable category, with 15.7% for the covalent (*n* = 109/695) and 20.0% (*n* = 68/340) for noncovalent inhibitors (Figure 9A1). The prevalence of adverse toxicological outcomes can be assessed using Pfizer’s 3/75 rule,^123^ where log *P* > 3 and TPSA < 75 Å^2^ are related to the adverse effect of chemical compounds. Application to our data set reveals that 2.4% (*n* = 17/695) of the covalent and 22.9% (*n* = 78/340) of the noncovalent binders do not comply with the Pfizer 3/75 rule, meaning that they may exhibit increased toxicity (Figure 9A2). It is worth highlighting that the Pfizer 3/75 rule does not take into consideration the possible presence of mutagenic functional groups. For instance, nitro groups are often present in DprE1 covalent inhibitors, although drugs containing nitro groups have been linked to mutagenicity and genotoxicity.^149^ It is worth noting that various reports indicate that nitro-containing DprE1 inhibitors exhibit favorable metabolic, microsomal, and plasma stability and reduced toxicity.^37^

#### hERG Inhibition

Throughout the drug development process, one of the most common undesirable side effects that contributes to a medicine’s failure is cardiac arrhythmias.^150^ Numerous forms of cardiovascular toxicity must be taken into account, as the promiscuous blocking of hERG cardiac potassium channels by small molecules poses a significant therapeutic challenge, with severe consequences for human health.^151−153^

The model implemented in StarDrop v7.2.0.32905 predicts that covalent DprE1 inhibitors exhibit the highest potential for hERG inhibition, with a mean pIC_50_ value of 6.16, with values ranging from 5.01 (10th percentile) to 7.21 (90th percentile) for most drugs, while the noncovalent binders varied from 4.28 (10th percentile) to 6.30 (90th percentile), with a lower mean of 5.26. In general, an experimental binding assay is indicated if the score is larger than 5, since the molecules are likely to display some toxicity linked to these cardiac potassium channels.^151−153^ We present a categorization histogram (Figure 9B) where it is shown that 90.4% of the covalent and 55.0% for the noncovalent inhibitors have pIC_50_ > 5. Both of these subsets also scored 58.7% (Cov) and 25.0% (NCov) of compounds with pIC_50_ > 6. Within the covalent inhibitors, 99.3% of the benzothiazinones have a pIC_50_ > 5, with 70.7% for pIC_50_ > 6 and 28.6% between 5 and 6. hERG inhibitions in BTZ have been observed previously, and further optimizations through SAR studies of three moieties (benzene ring, linker, and N-heterocycle) on the C-2 side chain of the BTZ scalffold have been performed, allowing identification of new lead compounds with reduced hERG liability (inhibition rate (IR) < 50% at 10 μM) without sacrificing antimycobacterial potency.^49,54^ The DNB class showed a smaller proportion for a predicted pIC_50_ > 6 (6.9%). A pIC_50_ of 5–6 is predicted for 100% of the inhibitors in both NQ and NTZ classes, followed by 68.2% for NBTO and 38.2% for DNB. Values of pIC_50_ below 5 are predicted for DNB (54.9%) > NBTO (31.8%) > BTZ (0.8%) inhibitors. Among the covalent binders, the NBTO class emerges as the one with lower predicted hERG inhibition. Predictions within the noncovalent inhibitors are pIC_50_ > 6, NC BTZ (81.8%) > 2-S-BTO (80%) > PTD (75%) > MP (65.4%) > CD (59.1%) > PP (55.6%) > 4-AQ (50%) > NHMS (20%). For pIC_50_ values in the range 5–6: AVMT, BI (100%) > BD (80%) > NHMS (60%) > AZA (57.9%) > 4-AQ (50%) > PP (44.4%) > CD (40.9%) > 2-CQ (37.5%) > MP (34.6%) > PTD (25%) > NAHPC (22.2%) > 2-S-BTO (20%) > NC BTZ (18.2%) > TPA (9.0%) > HYD (5.3%) > BTO (3.7%). pIC_50_ values below 5 are predicted for BTO (96.3%) > HYD (94.7%) > TPA (91.0%) > NAHPC (77.8%) > 2-CQ (62.5%) > AZA (42.1%) > BD, NHMS (20%).

1,4-Azaindoles scored 57.9% for pIC_50_ in the range 5–6 and 42.1% for pIC_50_ < 5. Reported hERG assays for this scaffold have shown no inhibition of the hERG channel at up to 33 μM (pIC_50_ < 4.48) concentrations,^77,79^ displaying a calculated absolute error (*x̅* ± σ) of 0.40 ± 0.11 log IC_50_ comparing to the predicted values (Table 5). Although the hydantoin heterocycle is linked to potential cardiotoxicity,^90,91^ predictions for this scaffold point to 5.3% with pIC_50_ between 5 and 6 and 94.7% with pIC_50_ < 5. The calculated absolute error (*x̅* ± σ) for the HYD class between the experimental and predicted data was in the range of 0.26 ± 0.14 log IC_50_, for the HYD compounds (Table 5). Thiophene-arylamide compounds showed a high proportion of predicted pIC_50_ < 5 (91.0%), which is in keeping with literature reports. In contrast, selected TPA compounds exhibited low inhibition profiles of the hERG channel (IC_50_ > 20 μM (pIC_50_ < 4.70)) across the series, indicating a low risk of blocking the cardiac potassium channel and causing QT prolongation.^98^ The calculated absolute error (*x̅* ± σ) between the experimental and predicted data was in the range of 0.26 ± 0.14 log IC_50_, for the TPA compounds (Table 5). Predictions for the BI series placed 100% of the compounds within a pIC_50_ range of 5–6, though hERG channel assays indicated no major safety liabilities, with values of IC_50_ > 33 μM (pIC_50_ < 4.48).^81^ The calculated absolute error (*x̅* ± σ) between the experimental and predicted data was in the range of 0.80 ± 0.11 log IC_50_, for the BI class, displaying the highest relative error (15.1 ± 1.8%) while using the predictive model (Table 5). For the benzothiazole group, an hERG assay showed that **TCA1** has no activity at IC_50_ > 30 μM (pIC_50_ < 4.52),^72^ in keeping with the prediction of 96.3% for BTO, for pIC_50_ values below 5. For an evaluation of the prediction model between the noncovalent binders, it shows that it seems to vary between the different scaffolds, with the HYD class having the best predicted values and BI the poorest.

**Table 5:**
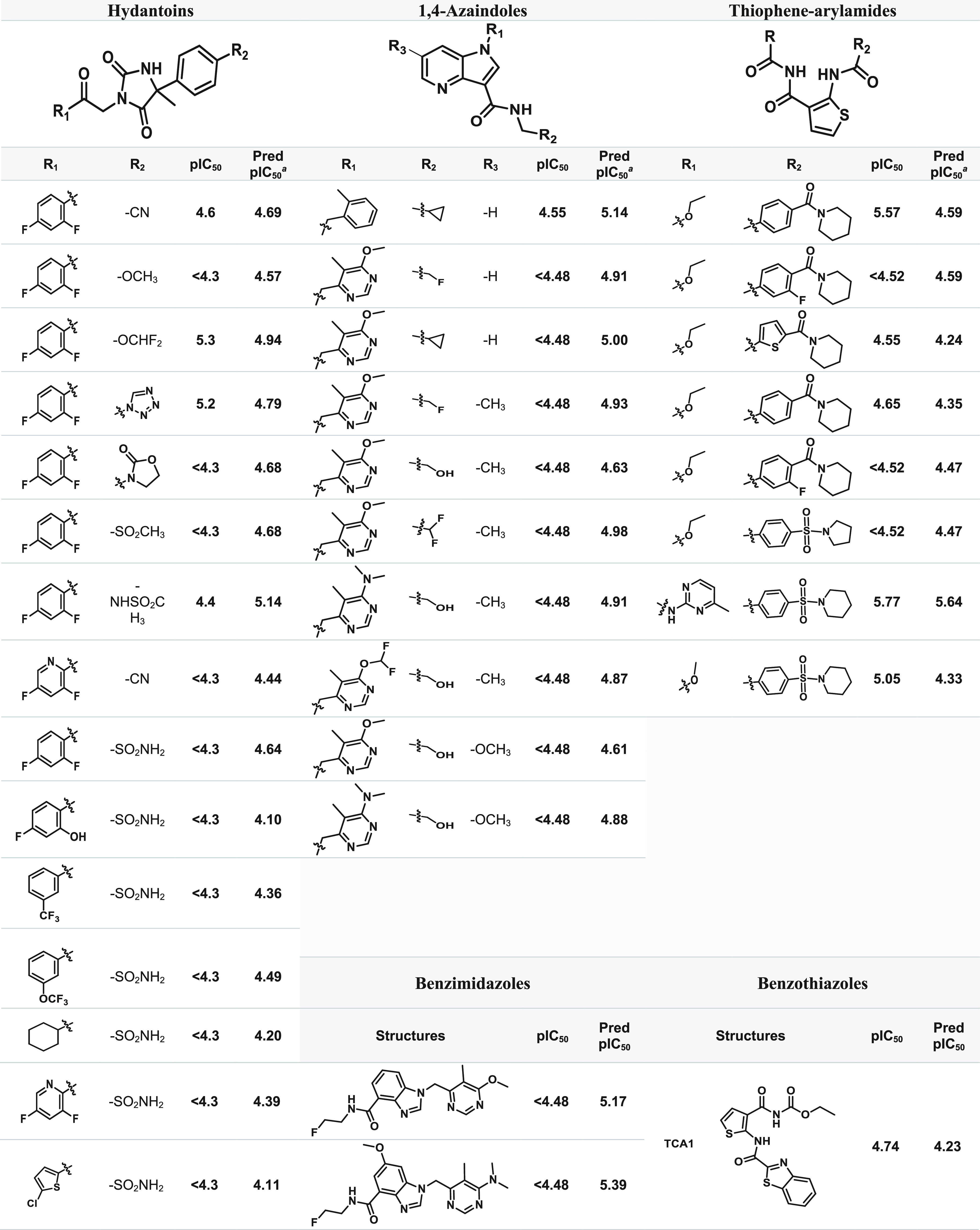
Experimental and Corresponding Predicted hERG pIC_50_ Data of the Noncovalent Inhibitors

a Pred pIC_50_: predicted hERG pIC_50_ computed by StarDrop v7.2.0.32905.

### AMES Mutagenicity

The AMES test is a biological assay used to determine the mutagenic potential of a chemical compound,^154,155^ which entails the activation of promutagens via mammalian metabolism.^156,157^ AMES mutagenicity predictions were computed using StarDrop modules’ toxicity models. The results yielded a high score of 96.0% (667/695) for covalent inhibitors, in contrast to the noncovalent binders, with only 32.5% (109/340) with an AMES positive prediction. This high value computed for covalent inhibitors was somewhat expected, since nitro-aromatics are generally associated with mutagenicity^149,158^—the nitro-aromatic moiety is a common motif in covalent DprE1 inhibitors. The results revealed predictions of AMES mutagenicity for NBTO and NQ (100%), followed by BTZ (98.8%) > DNB (80.4%) and, last, of the nonmutagenic nature of NTZ (0%). All tested experimental NBTOs were found to be AMES positive,^65^ agreeing with the predictions. This was rectified by the addition of a methyl group, adjacent to the nitro group of the NBTOs, affording the crowded benzothiazoles (cBTs), which tested AMES negative. Although experimental work has shown no indication of mutagenic or nitrosactive gene expression profiles following treatment with BTZ043, chemical proteomics showed evidence for induction of 60 genes, which was expected, as BTZs specifically target cell wall biogenesis. Therefore, concerns on the mutagenicity of the nitro group proved unfounded.^69^ The AMES test demonstrated that the DNPT did not generate mutations in *S. typhimurium* TA98 and TA100 strains, even with metabolic activation.^101^ For the noncovalent data set, it scored for mutagenicity of 34.9%, with 2-S-BTO (100%) > HYD (76.3%) > MP (69.2%) > BD (60%) > BI (55.6%) > PTD (50%) > NC BTZ (36.4%) > AZA (31.6%) > PP (22.2%) > BTO (18.5%) > CD (15.9%) > TPA (10.4%) > 4-AQ, AVMT, NAHPC (0%) (Figure 9C).

### PAINS and Structural Alerts

**Figure 10:**
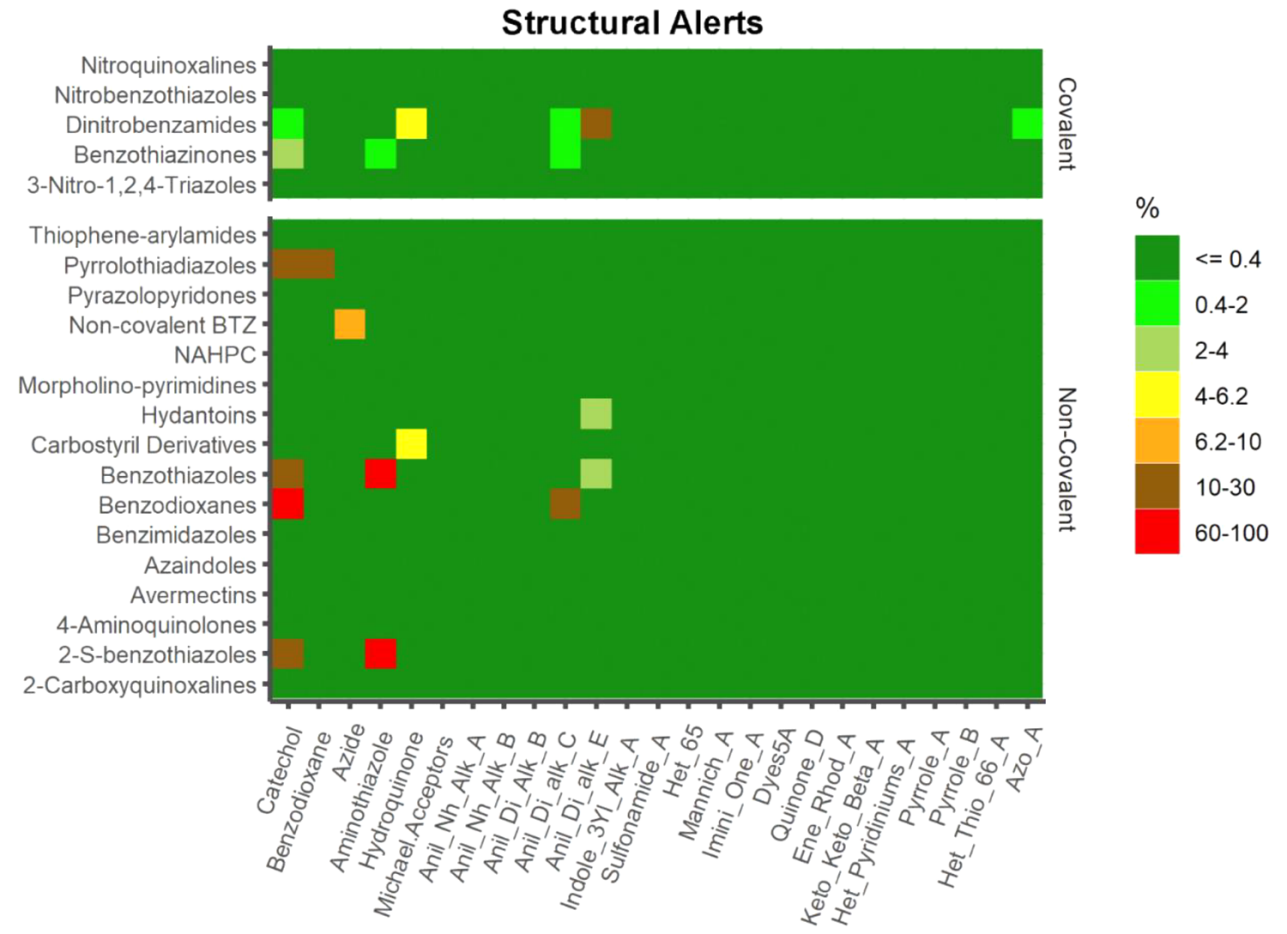
Matrix plot of structural alerts computed by ChemBioServer 2.0.

Substructural warnings have become a common feature of the triage process in biological screening campaigns to identify pan-assay interference compounds (PAINS). PAINS generate false-positive assay responses as a result of their reactivity under assay circumstances,^159^ which may include covalent modification, metal chelation, autofluorescence, aggregation, and redox reactivity, among others.^160−163^ Certain structural motifs (“structural alerts”) may result in covalent alteration of proteins or DNA, inducing negative effects (hepatotoxicity, CYP inhibition, *in vitro* genotoxicity, carcinogenicity).^160−163^ We screened our data set for PAINS count with StarDrop that embeds the original PAINS definitions, and we show that only 7.5% (52/695) of the covalent inhibitors scored for detected PAINS, with DNB having the highest percentage (29.4%), followed by the BTZ class (3.9%). The identified structural alerts (SA) for the covalent subset were Anil_Di_alk_E (3.45%) > catechol (2.01%) > hydroquinone (1.01%) > Anil_Di_alk_C (0.86%) > aminothiazole (0.43%) > benzodioxane, Azo_A (0.14%). The noncovalent set scored a higher proportion than the covalent set for PAINS, with 13.4% (45/340). The classes that contained SA were 2-S-BTO, BD (100%) > BTO (74.1%) > PTD (25%) > NC BTZ (9.1%) > CD (4.5%) > HYD (2.6%). Aminothiazole (11.48%) was the most frequent SA detected, followed by catechol (3.29%) and some residual Anil_Di_alk_E and hydroquinone (0.60%), together with anil_Di_alk_C, azide and benzodioxane (0.30%) (Figure 10). It is important to emphasize that, as for PC descriptors, PAINS substructure searches must be used cautiously when picking candidates, as there have been numerous observed deviations to these principles.

## Conclusions

DprE1 has been established as a potential therapeutic target for inhibiting mycobacterial cell wall biosynthesis, in which this enzyme is a highly druggable target against *M. tuberculosis*, and various chemical scaffolds have been developed since its discovery. Twenty-three distinct scaffolds have been found to exhibit a high affinity for this enzyme, with varying antimycobacterial activity and DMPK profiles, and these inhibitors are divided into covalent and noncovalent binders.

The design of DprE1 inhibitors can be challenging; therefore, prediction of PC descriptors and ADMET properties for these molecules may aid in the design of new lead compounds. An extensive PC descriptor analysis indicates that for inactive covalent DprE1 it may be necessary to optimize the compounds by increasing MW, *C* log *P*, *C* log *D*, HBA and TPSA, while reducing log *S* and HBD to match the active set’s corresponding properties more closely. In contrast, for inactive noncovalent DprE1 inhibitors it may be required to optimize the compounds by increasing MW, HBA, HBD, TPSA, FInd, and ROTBS. All these changes are likely to enhance the enthalpic component of drug binding through enhanced hydrogen-bonding contacts with the enzyme. Covalent DprE1 inhibitors tend to violate the Ro5 more frequently than the noncovalent counterparts. However, only a small proportion fails the criteria of two or more violations, indicating that the DprE1 inhibitors are more likely to have membrane permeability and hence be more readily absorbed in the human digestive system via passive diffusion. Almost all DprE1 inhibitors were predicted to have no CNS penetration, with the entire covalent subgroup scoring no CNS penetration and a residual value for noncovalent binders, reducing the possibility of side effects on the CNS. On the other hand, DprE1 inhibitors, particularly covalent binders, may act as P-gp substrates, which must be closely evaluated during drug optimization.

CYP3A4 was the major predicted isoform to metabolize DprE1 inhibitors, followed by the isoforms 2D6 > 2C19 > 2C9 for the covalent inhibitors and 2C9 > 2D6 > 2C19 for the noncovalent inhibitors. These predictions contribute to our understanding of the role of the CYP superfamily in the metabolic stability of DprE1 inhibitors.

Toxicity end points were also examined, and the cardiovascular toxicity of the DprE1 inhibitors *via* hERG inhibition was observed to be higher in the covalent than in the noncovalent subset, this observation holding for a cardiotoxicity investigation. Experimental data show that optimizations can be made to improve this feature, as seen in the case of the hydantoin class. It is worth noting that other data with BTZ and TPA have shown no inhibition of the hERG potassium channel. Covalent inhibitors have scored in a higher proportion for mutagenic warnings than the noncovalent binders. This computed high value was expected, since nitro-aromatic molecules are known to be mutagenic. In terms of undesirable structural motifs (structural alerts and PAINS), DprE1 inhibitors have a small number of these substructures, with the noncovalent set scoring higher for PAINS than the covalent set.

In conclusion, several molecular properties that should facilitate the design and optimization of future DprE1 inhibitors were described, allowing for the development of novel compounds targeting *M. tuberculosis*. As a mere aside, we wish to emphasize that our study comparing predicted and experimental values reveal that software tools employed to predict specific DMPK parameters must be used with caution while optimizing a drug class.

## Supporting Information Available

Molecular formula strings (SMILES) and extensive data for 1519 DprE1 compounds in 57 variables (XLSX)
